# Thomas F. Rochester, M. D.

**Published:** 1886-03

**Authors:** 


### Thomas F. Rochester, M. D.

During the last few months, Dr. Rochester has suffered a painful and prolonged illness. Reports have been circulated from time to time that have caused his many and numberless friends great anxiety. Dr. Burwell, his attending physician, has always been hopeful of him, and has quieted the fears of the medical men who have gone to him fearing the worst. At this writing, however, we are glad to be able to report that the Doctor's complete recovery is an assured fact. This news will be joyfully received by all our readers. That we may not have our hope again dispelled, and that he may go on to speedy recovery, is the earnest and fervent wish of each one of us.

Dr. Rochester was born at Rochester, N. Y., October, 1823, consequently he will soon be sixty-three years of age. He received his collegiate education at Geneva College, where he graduated in 1845. In 1848 he graduated, at the age of twenty-five, from the University of Pennsylvania. For a year he was Assistant at Bellevue Hospital, New York. On completing his term of service he went to Europe, where he remained a year or two in the pursuit of knowledge. He returned to this country and established himself in New York City. He must have taken a high position among his professional brethren to have had the chair of the already illustrious Flint offered him after two years. He accepted the Chair of Medicine in the Buffalo Medical College, and has occupied it ever since. He has labored long and diligently for the success of his school. He has always shown an interest in his graduates, as well as in many deserving young men who have come from other schools and with whom he had become acquainted. One of the many characteristics of his broad and generous nature, is kindness and generosity to the younger physicians.

The Doctor is a member of the State Society, but does not approve of the new Code adopted by that organization, and was one of the prominent physicians who aided in organizing the present State Medical Association. He is a member of the Erie County Society, of the Buffalo Medical and Surgical Association, and has been the President of both. During these later years, as well as earlier, he has been a constant attendant on these meetings, and has, by his intelligent and ready discussions of the various topics presented from time to time, added much to his reputation.

In the charitable work of our city he has always taken an active part. For many years he has occupied the position of attending or consulting physician to the Buffalo General Hospital, and for twenty years was attending physician to the Sisters of Charity Hospital. This amount of gratuitous work must necessarily prove a great burden to any physician who has had such a large private practice, but he was known as one of the most regular and most devoted physicians of the staff. This is not the end of his charitable and humanitarian work. He is prominent in everything which will advance the interests of our city. In church and in social life he is equally prominent; in the sick-room, kind and sympathetic, as well as skillful. We can only add that such a record as this can well be looked to with pride by all his friends. It can well be the example for any young man.

We are certainly rejoiced that in a few short weeks he will be able to resume his practice, that his wise counsel will soon be heard in the Society, and his kind and benevolent face will soon be seen among us.

